# Assessing the Capacity of Plant Species to Accumulate Particulate Matter in Beijing, China

**DOI:** 10.1371/journal.pone.0140664

**Published:** 2015-10-27

**Authors:** Li Mo, Zeyu Ma, Yansen Xu, Fengbin Sun, Xiaoxiu Lun, Xuhui Liu, Jungang Chen, Xinxiao Yu

**Affiliations:** 1 College of Soil and Water Conservation, Beijing Forest University, Beijing, China; 2 College of Forestry, Beijing Forestry University, Beijing, China; 3 College of Environmental Engineering, Beijing Forestry University, Beijing, China; Institute of Botany, CHINA

## Abstract

Air pollution causes serious problems in spring in northern China; therefore, studying the ability of different plants to accumulate particulate matter (PM) at the beginning of the growing season may benefit urban planners in their attempts to control air pollution. This study evaluated deposits of PM on the leaves and in the wax layer of 35 species (11 shrubs, 24 trees) in Beijing, China. Differences in the accumulation of PM were observed between species. *Cephalotaxus sinensis*, *Euonymus japonicus*, *Broussonetia papyrifera*r, *Koelreuteria paniculata* and *Quercus variabilis* were all efficient in capturing small particles. The plants exhibiting high amounts of total PM accumulation (on leaf surfaces and/or in the wax layer), also showed comparatively high levels of PM accumulation across all particle sizes. A comparison of shrubs and trees did not reveal obvious differences in their ability to accumulate particles based on growth form; a combination of plantings with different growth forms can efficiently reduce airborne PM concentrations near the ground. To test the relationships between leaf traits and PM accumulation, leaf samples of selected species were observed using a scanning electron microscope. Growth forms with greater amounts of pubescence and increased roughness supported PM accumulation; the adaxial leaf surfaces collected more particles than the abaxial surfaces. The results of this study may inform the selection of species for urban green areas where the goal is to capture air pollutants and mitigate the adverse effects of air pollution on human health.

## Introduction

Particulate Matter (PM), a common air contaminant, has become one of the most important atmospheric pollutants worldwide. Power stations, vehicle exhaust, cement factories, and industrial processors emit primary PM directly. Secondary particle reactions generate secondary PM in the atmosphere through the formation of substances such as nitrogen oxides (NO_x_), sulfur dioxide (SO_2_), ammonia, and volatile organic compounds (VOCs) [1,2]. Harmful PM, especially PM2.5 (made up of particles less than 2.5 micrometers in diameter), often contains highly toxic components, such as heavy metals, polychlorinated biphenyls, polycyclic aromatic hydrocarbons, and other potential carcinogens [3,4,5]. Pope et al. [6] stated PM in the atmosphere carries a risk of creating harmful effects to human health, such as lung cancer and cardiopulmonary disease. Particulates are of particular concern in urban and industrial areas where levels often exceed allowable limits. Children may be especially vulnerable to air pollution [7].

Numerous studies have demonstrated that plants can purify air by absorbing atmospheric pollutants, and may significantly affect air quality [8]. For example, urban vegetation can decrease PM concentrations in the atmosphere; one study estimated that 772 tons of PM10 were removed by trees during one year in the city center of Beijing, China [9]. In cities of the United Kingdom (UK), McDonald et al. [10] found that planting trees in one-quarter of an urban area could reduce PM10 concentrations by 2–10%. A study in Chicago, IL, USA, suggested that if urban trees occupied 11% of the city area, they would remove approximately 234 tons of PM10 every year [11]. Nowak et al., concluded that urban vegetation removed approximately 215,000 tons of PM10 per year across the United States in 2006 [12].

The surfaces of leaves and bark of vegetation accumulate PM. Air turbulence in tree crowns increases the deposition of PM on leaves. Trees collect more pollutants, including coarse and fine PM, than shorter vegetation [13]. Species with trichomes and/or rough leaf surfaces are considered more effective accumulators of PM [14, 15]. Another study showed that broad-leaved species with rough leaf surfaces accumulate PM more effectively than those with smooth surfaces [16]. In addition, needles of coniferous trees, which have a unique microstructure and produce a thicker epicuticular wax layer, capture PM more effectively than broad-leaved species [17].

The species and planting systems that are most efficient in removing PM should be selected to maximize the benefits of urban vegetation in improving air quality. While much is known about the mechanisms involved in the deposition of PM on vegetation [18], fewer studies have addressed the differences in PM accumulation of various species, particularly for the smallest fractions (PM2.5). Given the large number of tree, shrub, and cultivar options available, species-specific information could help land managers select the optimal vegetation for urban and suburban areas.

To optimize the selection of beneficial vegetation in different urban settings, Dzierżanowski et al. and Escobedo et al. [19, 20] found that evaluating the ability of different species to accumulate and retain PM and air-borne pollutants was very useful. Furthermore, gaining a better understanding of the mechanisms involved in the capture of size-fractionated particles both on the surfaces and in the wax of different species would be beneficial. This study explores these topics.

## Materials and Methods

### Plant material and field conditions

This study involved plant materials collected from trees and shrubs on the campus of Beijing Forestry University, Beijing, China. As the capital of China, Beijing serves as a political, economic, and cultural center that appropriately represents the air quality of northern urban cities in China. Fewer vehicles visit the campus than traverse the main streets of Beijing, and the campus has more vegetation than other areas. The campus is generally considered rural, and is protected from traffic or industrial pollution.

The plants studied for this project are all commonly planted in northern China (Table 1). Samples were collected randomly across the relatively small campus; any small environmental variations on campus were not expected to influence the results. The sampled plants were in good condition, with little or no disease and/or pests. Weather conditions before and during each sampling event were similar and stable across all sampling points. When the wind speed is less than 5 m/s, it has no obvious effect on particles which are accumulated on the leaf [21]. Sample dates were selected when no precipitation had occurred during the week before sampling and during time periods following a week with very little wind. For each species, samples were collected from the same plant height, and from the same direction (e.g., north or south facing leaves) to avoid any influence from wind. Four individuals were sampled for each species.

**Table 1:** Results of species clustering analysis related to leaf surface PM, in-wax PM and total PM as variables. Cluster 1 and 3 had the smallest and largest quantities of captured PM, respectively.

**Classification**	**Species**	**Cluster**
Shrub	*Paeonia suffruticosa* Andrews	1
	*Euonymus japonicus* Thunb.	2
	*Cephalotaxus sinensis* (Rehder et E. H. Wilson)H. L. Li	3
	*Malus* × *micromalus* Makino	2
	*Chimonanthus praecox* (L.) Link	2
	*Amygdalus triloba* (Lindl.) Ricker	2
	*Syringa oblata* Lindl.	2
	*Weigela florida* (Bunge) A. DC.	1
	*Kolkwitzia amabilis* Graebn.	2
	*Philadelphus pekinensis* var. *kansuensis* Rehder	1
	*Prunus Cerasifera* Ehrh.	2
tree	*Rhus typhina* L.	1
	*Koelreuteria paniculata* Laxm.	2
	*Platanus occidentalis* L.	3
	*Morus alba* L.	2
	*Fraxinus pennsylvanica* Marsh.	1
	*Ginkgo biloba* L.	2
	*Ulmus pumila* L.	1
	*Salix matsudana* Koidz.	1
	*Broussonetia papyrifera* (L.) L'Hér. ex Vent.	3
	*Quercus variabilis* Blume	3
	*Acer truncatum* Bunge	1
	*Metasequoia glyptostroboides* Hu & W.C. Cheng	2
	*Syringa reticulata* ssp. *Amurensis* (Rupr.) P.S. Green & M.C. Chang	2
	*Aesculus chinensis* Bunge	2
	*Toona sinensis* (A. Juss.) Roem.	1
	*Tilia tuan* Szyszyl.	1
	*Populus* × *canadensis* Moench	1
	*Catalpa speciosa* (Warder) warder ex Engelm.	1
	*Ailanthus altissima* (Mill.) Swingle	1
	*Euonymus bungeanus* Maxim.	1
	*Sophora japonica* L.	1
	*Fraxinus mandschurica* Rupr.	1
	*Crataegus pinnatifida* Bunge	2
	*Gleditsia sinensis* Lam.	1

### Sample collection and analysis of PM

All sampling was conducted during April, 2014. Samples from 35 species, including 11 shrubs and 24 trees, were gathered on the same day at the beginning of the growing season. For each species, all leaf samples were collected from a single individual, placed in plastic bags and sealed, labeled with serial numbers, and stored at 4°C in a clean laboratory refrigerator prior to analysis. PM collected from the leaf surfaces and in the leaf waxes was analyzed using the methods of Dzierżanowski et al. (2011) [19].

Filters were weighed before and after particulate removal and sorted using a balance (BT125D, Sartorius Co., Ltd., Beijing, China) sensitive to 0.00001 g. Filters were passed through a deionizer gate (AP-BC2451, AP&T, Shanghai, China) to avoid an electrostatic charge during filtering. Then the filters were placed in a polytetrafluoroethylene (PTFE) balancing box under the constant temperature (25°C) and constant humidity (40%) for 48 hours. The size of the balancing box is 80cm*80cm*80cm. A balance and a humidity controller (WHD48-11, ACREL Co., Ltd., Jiangsu, China) were placed in the balancing box. Filters were used for both the water and chloroform fractions (used to remove PM from leaves and wax, respectively; EMD Millipore Corp., Billerica, Massachusetts, USA). The filters facilitated three sequential levels of particulate separation: fine, coarse, and large particles with diameters of 0.2–2.5 μm, 2.5–10 μm and those > 10 μm, respectively. In this study, we did not measure the PM0.2 (diameter < 0.2 μm), because after using an LDSA (Mastersizer 2000, Malvern, England, measurement range: 0.02–2000 μm) in the pre-experiment, no PM0.2 was found on the leaf surfaces.

This filtering and weighing method limited the PM size range to the pore size of the filters. In this study, PM detection was limited to PM sizes with diameters of 0.2–100 μm, both on the leaf surface and in the wax layer.

Each washed leaf sample was scanned using a scanner (HP Scanjet 4850, China Hewlett-Packard Co.,Ltd.,Beijing, China), then the area measurements were calculated using Photoshop CS6 software. Although PM was washed from the adaxial and abaxial surfaces of leaves, the mass obtained was expressed in per unit area per leaf. This allows us to distinguish between PM on the surface and in the waxes, and allows us to calculate the amount of total PM of the different size fractions, summarized for surfaces and waxes.

To test the relationships between leaf traits and PM accumulation, typical leaf samples for each selected species were studied using a scanning electron microscope (SEM) (S-3400N II, Hitachi Japan Co., Ltd., Tokyo, Japan). Leaf samples were collected at the same time from the same plants as for the samples used in the filtering experiments above. Samples were stored in paper bags and dried in a dryer (DHG-9145A, Shanghai Yiheng Scientific Instrument Co., Ltd.,Shanghai, China) at 60°C for 48 hours. Micrographs were taken at two different magnifications (×420SE, ×2.10k SE).

### Statistical analysis

K-means clustering using SPSS18.0 was used to categorize the ability of different species to accumulate PM. Three sets of species were identified based on their ability to accumulate PM: low, intermediate or high. Clustering was run for each sample using standardized average accumulation for the six PM size fractions (fine, coarse and large PM accumulation for both leaf surface and in-wax particles).

One-way analysis of variance (ANOVA) was used to test for differences in PM accumulation among species. Tests were performed separately for trees and shrubs. Discriminate analysis was used to visually observe differences between trees and shrubs, and the distribution of PM on individuals. Multiple comparisons among species were performed using Least-Significant Difference multiple comparison tests. Values presented on the figure bar charts display means ± SD, *n* = 4. The figures were calculated using Origin 8.0 (Microcal, Northampton, MA, USA).

## Results and Discussion

### Overall Description

Plant species differed in the accumulation of PM fractions (large, coarse, and fine) on leaf surfaces and in waxes. Considerable differences were observed in PM deposition for most plant species. To better understand the abilities of different plants to accumulate PM, species were clustered into three groups, based on the sum of surface and in-wax PM of the three particle size fractions (Table 1). Cluster 1, 2 and 3 plants accumulated low, intermediate and high quantities of PM and included 17, 14 and four species, respectively.

**Fig 1:**
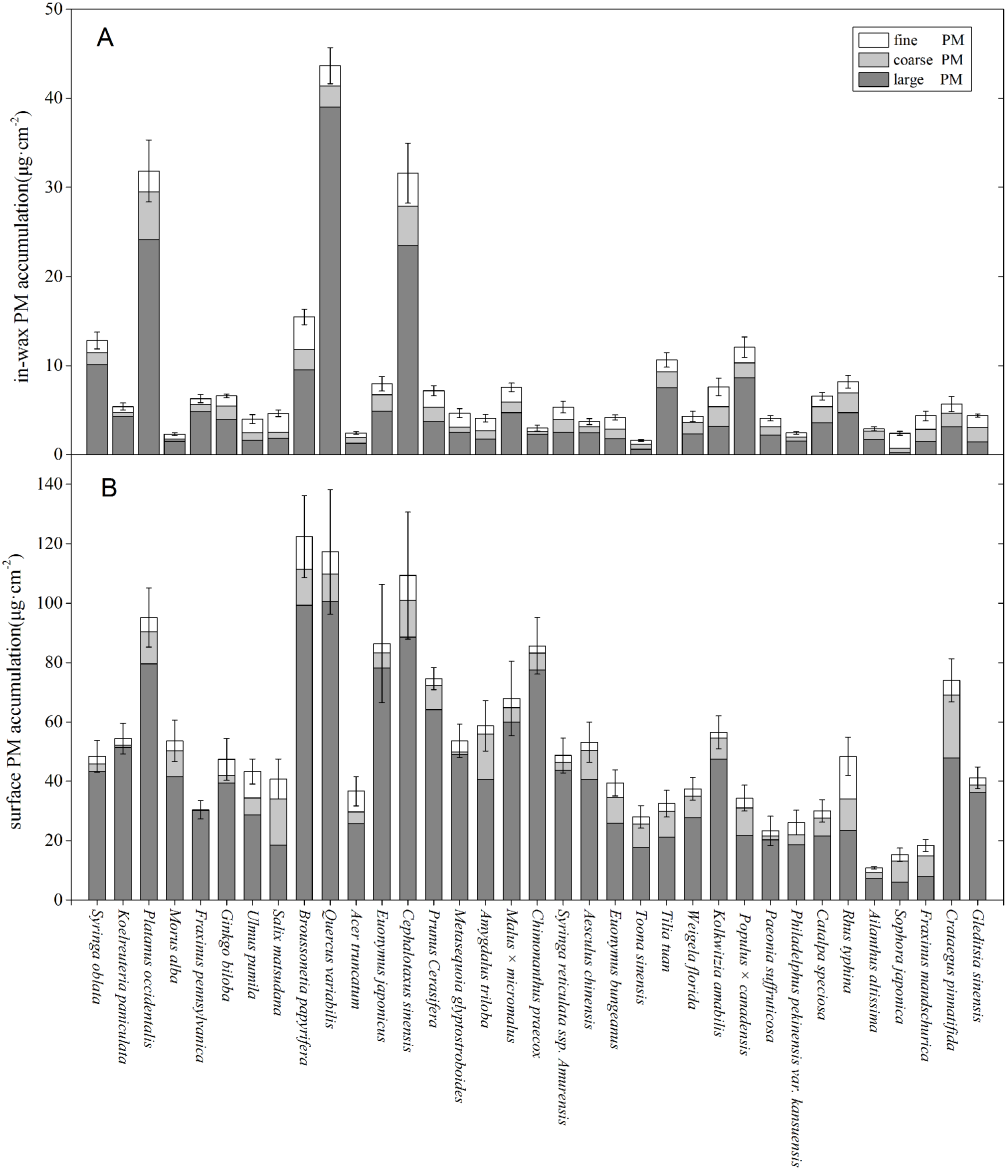
PM accumulation of different size fractions in-wax (A) and on leaf surfaces (B). Error bars are SE of total PM accumulation.

Fig 1 shows the concentrations of size-fractionated particles of 35 plant samples, including both surface and in-wax PM. Broad-leaved plants could accumulate PM significantly.*Platanus occidentalis*, *C*.*sinensis*, *Quercus variabilis* and *B*. *papyrifera* showed high total PM accumulation on the leaf surface and in the wax layer (127.0–160.9 μg/cm^2^). *Ailanthus altissima*, *Sophora japonica*, and *Fraxinus mandschurica* had considerably lower total surface PM accumulation (less than 20 μg/cm^2^). *Toona sinensis*, *Morus alba* and *Sophora japonica* had considerably lower total in-wax PM accumulation (< 2.5 μg/cm^2^). The average PM accumulation across all 35 species was 62.0 μg/cm^2^.

**Fig 2:**
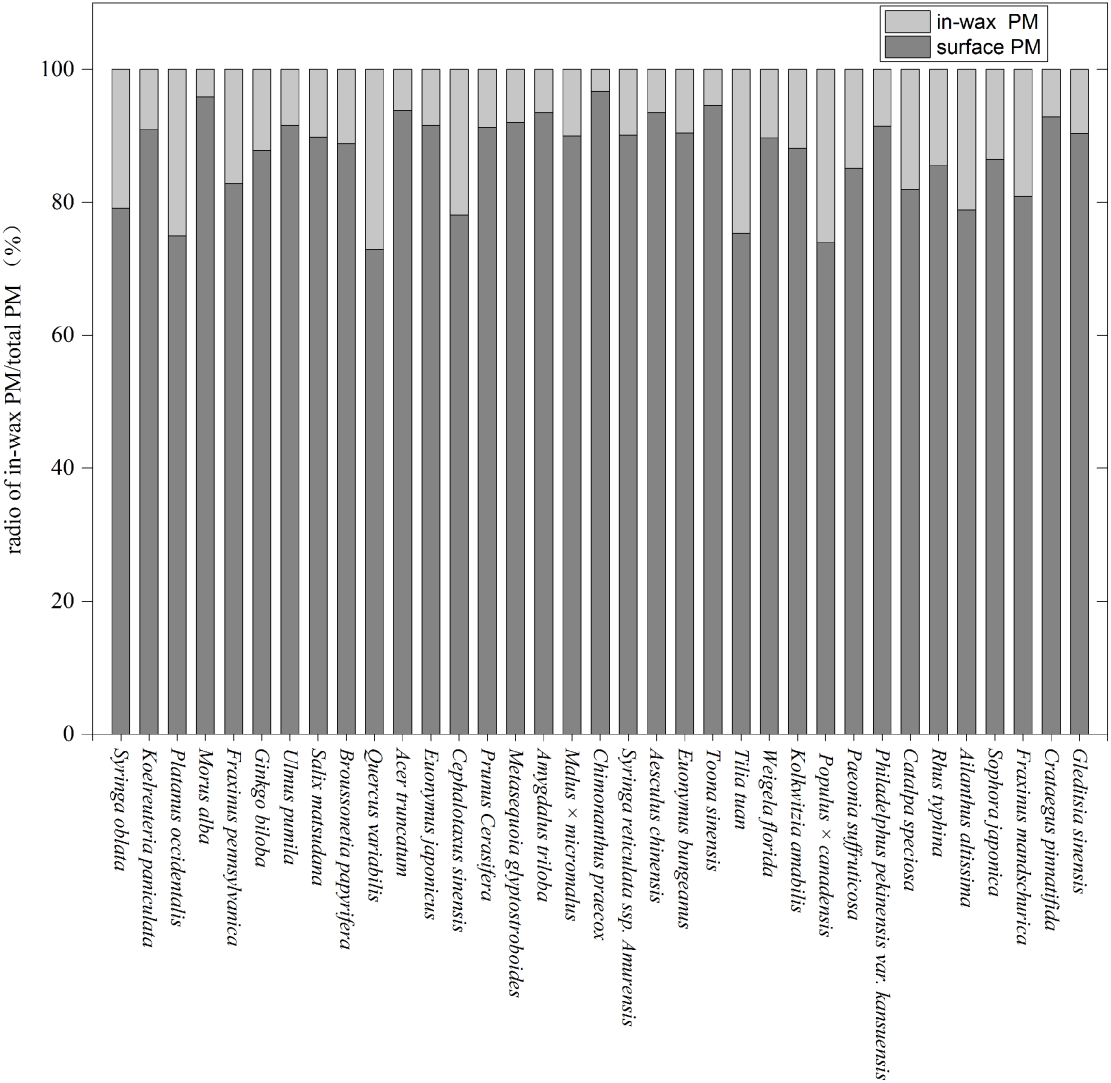
PM accumulation on leaves of 35 species presented as the ratio of in-wax PM to total PM.

The ratio between in-wax PM and total PM differed significantly between species (Fig 2). On average, across all 35 species, 12.84% of the PM deposition was in waxes on the leaf surfaces. One fifth of the 35 species had a ratio of in-wax deposits to leaf surface deposits between 5% and 15%. The lowest and highest ratios were found in *Chimonanthus praecox*(3.26%) and *Q*.*variabilis*(26.9%), respectively.

The structure and wettability of leaf blades influence their ability to intercept and fixed atmospheric PM and adsorb particles for several reasons as discussed below[22–25]. The leaf surface structures of each species are unique to that species, as discussed in detail below in the section subtitled “Importance of leaf traits.” Furthermore, the action of the particles themselves and the role of the blade influence PM accumulation. In the lower atmosphere, both fine and coarse particles are transported by turbulent eddies of mechanical and convective origin, while large particles are transported primarily by turbulence[26]. The ability of a species to retain PM depends in part on weather conditions and the dispersion of emissions within a basin. Wind may affect the PM previously deposited on surface of plants or rain may wash PM into the soil. For example, after wet or dry PM is deposited on leaves, that PM may become airborne again during a dry and windy period through resuspension. McPherson et al. [27] estimated that up to 50% of deposited particles may become resuspended. The fact that different plant species secrete different amounts of wax also leads to differences in the amount of in-wax PM that plants accumulate.

Currently, only a limited amount of research has been conducted related to the ability of several plant species to accumulate PM in China. This paper fills this gap in our knowledge of the ability of common plant species to accumulate different sized particles by testing a wide variety of common horticultural species typically used in urban greening projects in the Beijing area. Few studies have addressed the differences in PM accumulation in the wax layer of different species, particularly for size-fractionated particles. Having accurate data related to the amount of PM accumulated on plant leaves is helpful. Classification of the particles can clarify the ability of plants to adsorb different sized particles on leaf surfaces. The quality of atmospheric PM and its diameter is cube relationship; therefore, larger particles are normally high quality particles. The effects of small particles which often occur in large quantities may be overlooked and damage may occur if only the quality of particles is analyzed. Therefore, it is important to classify the particles.

For this study, leaves were collected at the beginning of the season, so the amount of accumulated PM does not reflect the total mass of deposited PM over the growing season. Future research should be conducted to study the accumulation of PM during the entire growing season.

### Differences between shrubs and trees

**Fig 3:**
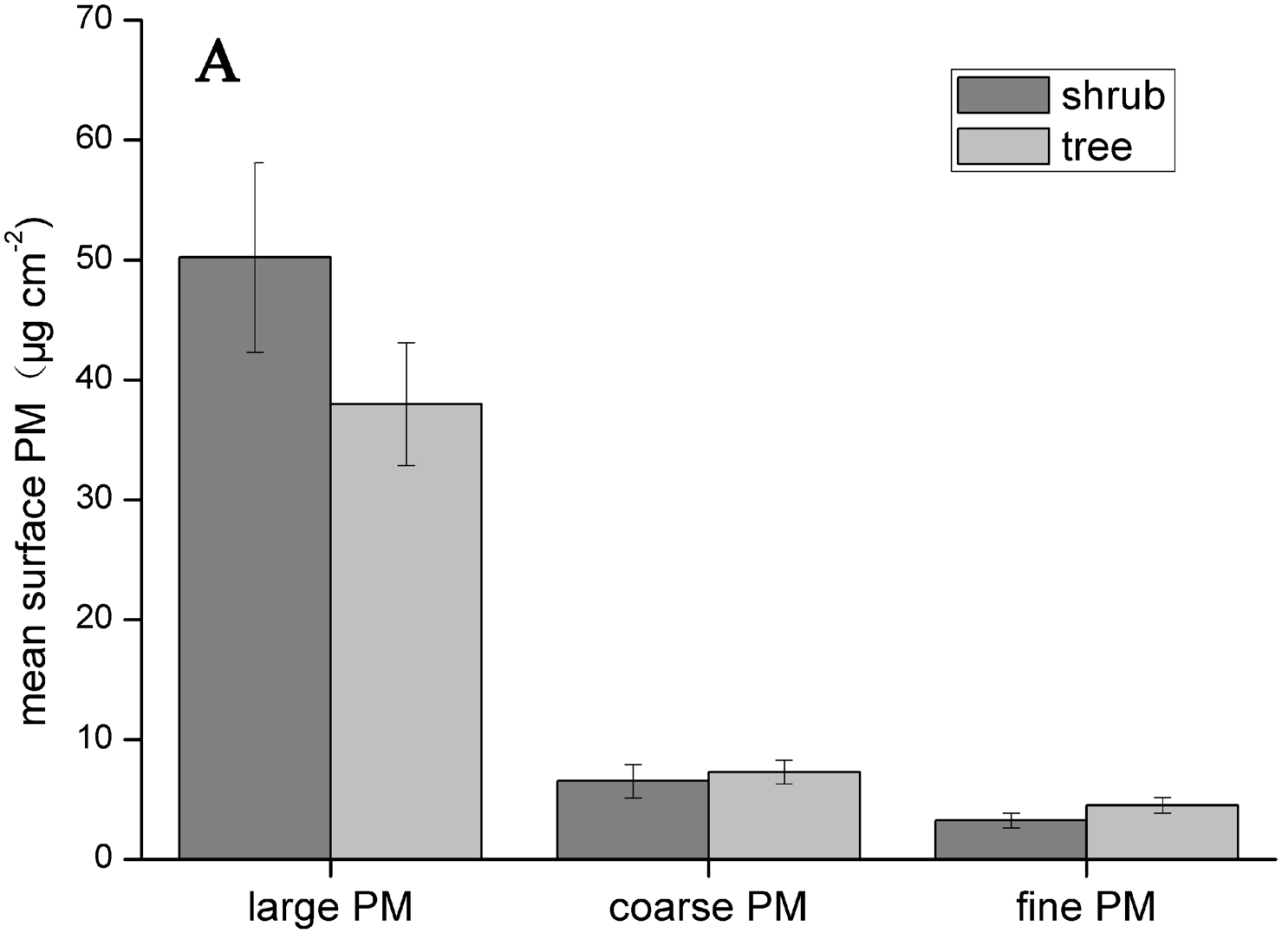
The average PM accumulation (large, coarse and fine) on shrub and tree leaf surfaces. Data are mean ± SE.

**Fig 4:**
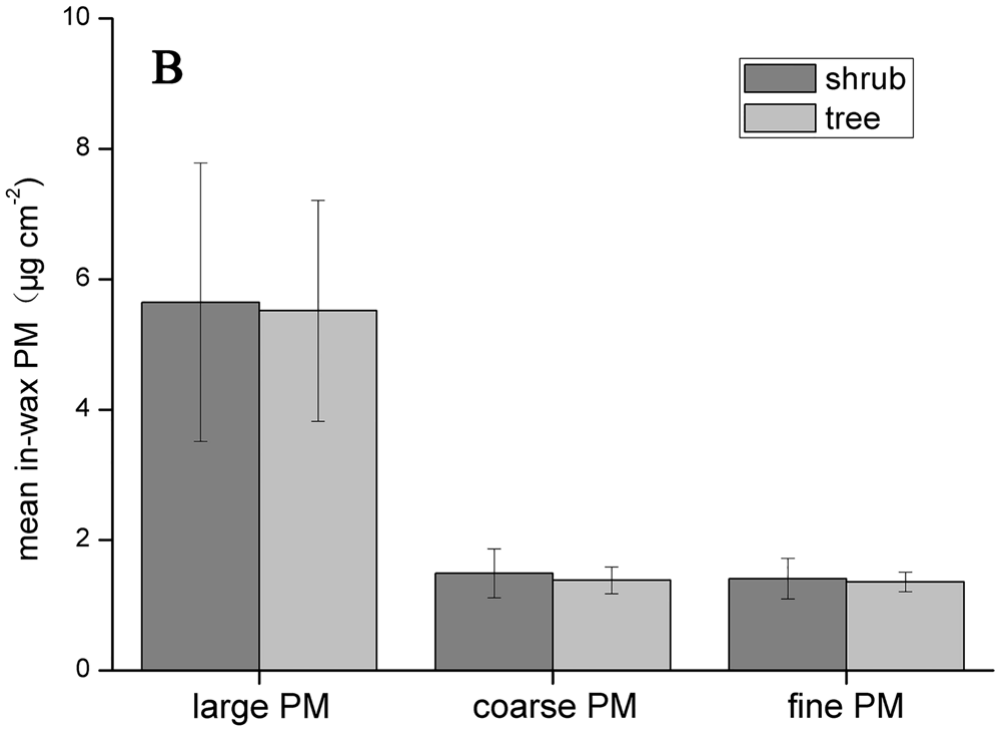
The average PM accumulation (large, coarse and fine) in shrub and tree wax. Data are mean ± SE.

Figs 3 and 4 show the mean PM accumulations on shrubs and trees, including the three PM fractions described above. Shrubs have a stronger ability to adsorb large particles onto their leaf surfaces than trees; conversely, smaller particles are more easily adsorbed by tree leaves. The amounts of size-fractionated particles in the wax layer were similar between trees and shrubs; trees were slightly better at adsorbing all particle sizes than shrubs.

**Fig 5:**
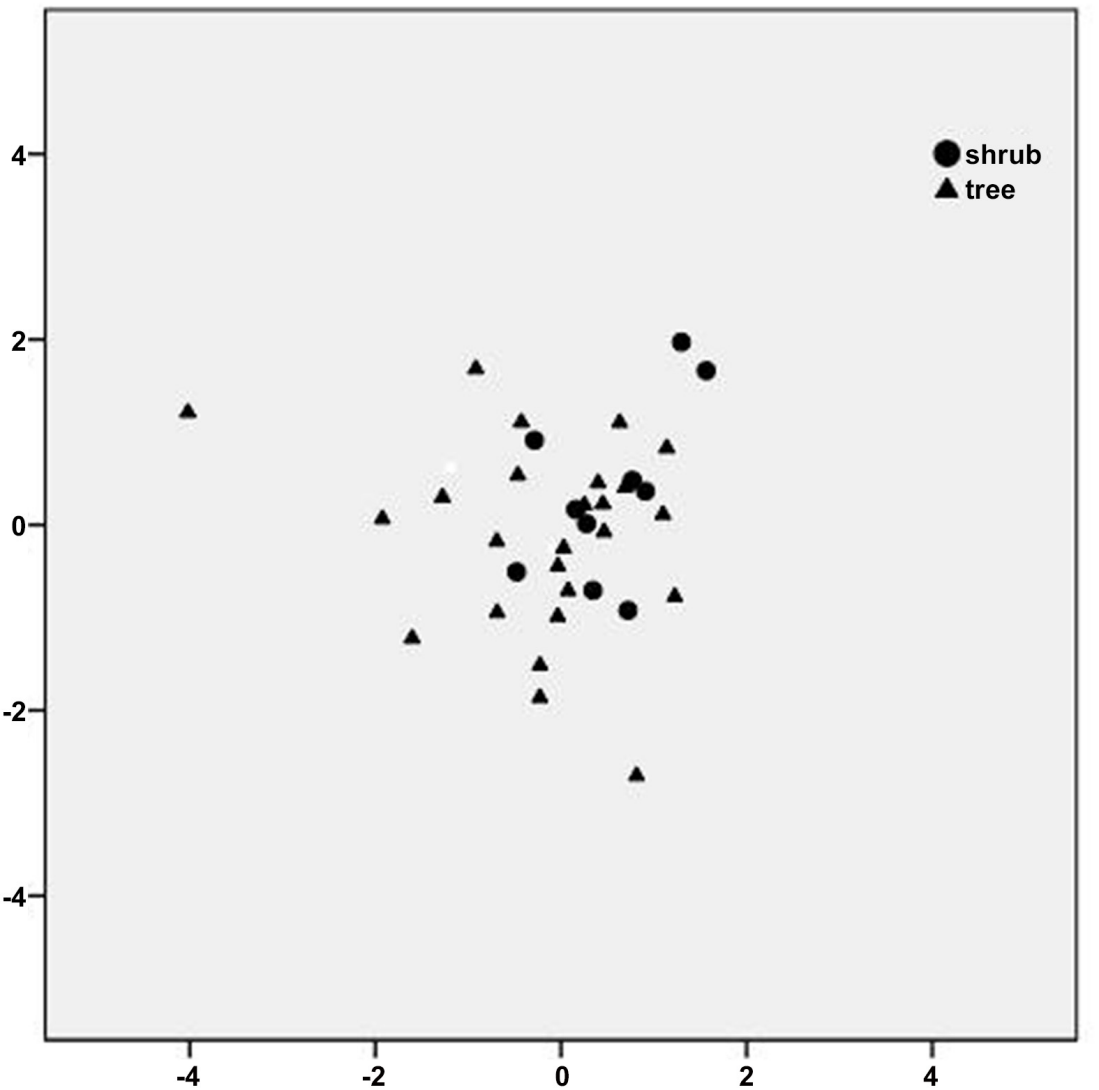
The discriminate analysis diagram of shrubs and trees.

Fig 5 shows the distribution of shrub and tree PM accumulation based on discriminate analysis; no significant differences were observed between trees and shrubs, indicating trees and shrubs do not significantly differ in PM accumulation. Because the number of sampled shrub (10) and tree (25) species differed in the present study, the statistical data for shrubs are not as representative as the data for trees. The single factor ANOVA revealed no statistically significant differences between shrubs and trees with respect to the PM deposition on leaf surfaces or in the wax layer, for each particulate size. However, the difference between shrubs and trees is greater for surface PM deposition than for in-wax PM deposition.

The presence of plants increases the roughness of the earth’s surface, resulting in lower wind speeds and allowing atmospheric PM to be deposited more easily. The crowns of trees are usually higher off the ground than those of shrubs. PM accumulation mainly comes from the precipitation of particles from the atmosphere. The large leaf area of some crowns allows them to accumulate larger amounts of atmospheric PM than trees with smaller leaf area [28,29]. Broad-leaved trees are usually considered to be effective accumulators of PM because of their large leaf area, broad crowns and high rates of PM absorption[30]. Shrubs are usually shorter than trees (1–2 m high) and are nearer to the ground; this proximity to the ground counteracts their lack of height, because they collect PM that exists closer to the ground that taller trees do not adsorb. This variation in plant height allows trees and shrubs to reduce PM on different spatial scales. As such, planting both trees and shrubs may efficiently reduce PM both near the ground and higher in the canopy.

### The situation related to size-fractionated particles

PM accumulation differences were also observed between species as Fig 1 showed. Across all plants, *Q*. *variabilis* (100.6±18.4),*B*. *papyrifera* (99.3±13.5), *C*. *sinensis* (88.6±17.7) and *P*. *occidentalis* (79.6±11.3)showed the highest large particle accumulation on leaf surfaces. *S*. *japonica* (6.08±1.08) and *A*. *altissima* (7.15±0.73) showed the least. The average large surface PM accumulation is 41.5μg/cm^2^.

For coarse particles, *Crataegus pinnatifida* (21.2±2.68), *Salix matsudana* (15.5±3.03), *Amygdalus triloba* (15.3±2.17) and *C*. *sinensis* (12.4±1.69) accumulated the most on leaf surfaces, while *Fraxinus pennsylvanica* (0.07±0.02) and *K*. *paniculata* (0.78±0.06) accumulated significantly lower amounts of coarse particles. The average coarse surface PM accumulation is 7.07μg/cm^2^.

*F*. *pennsylvanica* (0.13±0.08)also had the lowest amounts of fine PM deposition on leaf surfaces; *Rhus typhina* (14.28±2.66), *B*. *papyrifera* (10.9±1.67), *Ulmus pumila* (8.95±1.45) and *C*. *sinensis* (8.33±2.45) showed the most fine PM deposition. The average fine PM accumulation is 4.15μg/cm^2^.

*Q*. *variabilis* (39.0±2.29), *P*. *occidentalis* (24.2±2.92) and *C*. *sinensis* (23.4±2.14) had high particle accumulation in foliage wax, across all three particle sizes. *S*. *japonica* (0.23±0.03) and *T*. *sinensis* (0.61±0.07) showed low large particle accumulation in waxes*; C*. *praecox* accumulated considerably less coarse particles (0.29±0.05) and fine particles (0.41±0.16). Overall, the plants with high total PM accumulation (on leaf surfaces and/or in wax layer), showed comparatively high concentrations across all particle sizes.

When considering the accumulation of PM on leaf surfaces, only slight correlations were observed between the mass of large and coarse PM fractions, and only weak positive correlations were observed between the mass of large and fine PM fractions across species. The positive relationship between the mass of coarse and fine PM fractions was statistically significant (*P*< 0.05). The correlation coefficients were all positively statistically significant for in-wax PM accumulation at all three levels analyzed here across species (Table 2). Sæbø[31] found only weak to moderate positive correlations between the accumulation rates of different PM fraction on leaves; this agrees with the present study.

**Table 2:** ANOVA results showing the accumulation of different size PM fractions on tree and shrub leaves.

	Classification	Total PM		Large PM		Coarse PM		Fine PM	
		Mean (μg/cm^2^)	Sig.	Mean (μg/cm^2^)	Sig.	Mean (μg/cm^2^)	Sig.	Mean (μg/cm^2^)	Sig.
**Surface PM**	Trees	49.8		38.0		7.29		4.51	
			0.338		0.207		0.670		0.261
	Shrubs	60.0		50.2		6.52		3.25	
**In-wax PM**	Trees	8.26		5.52		1.38		1.36	
			0.956		0.966		0.791		0.873
	Shrubs	8.45		5.65		1.49		1.41	

In Qingdao, Shandong, China study, Li [32] observed that *P*. *occidentalis* accumulated the largest amount of PM on tree leaves of eleven species studied there, while *Fraxinus Pennsylvanica* accumulated the least PM. *Euonymus japonicus* was also the most effective shrub for accumulating PM. This study concluded that the largest amount of PM accumulated was 2.43-times the smallest amount accumulated. Wang [25] also observed that *P*. *occidentalis* and *E*. *japonicas* accumulated PM very well, similar to the results of our study. Despite the fact that leaves were collected in different places and in different seasons for different studies, all species mentioned above demonstrated an ability to accumulate PM and those species occur in most parts of China. However, Wang [25] found that *Ginkgo biloba* accumulated less PM than *Populus* × *canadensis*, which is an opposite finding from our study.

In light of those contrasting findings, very small differences in deposited mass clearly represent a large number of particles. As such, two possible reasons exist for the differences. First, the studies were conducted in different places. Wang studied plants in Xi’an [25]; ours was in Beijing. Second, although the leaves were all collected in April, the Xi’an leaves were mature, whereas the leaves in Beijing were still growing. The morphological structures also differed between the leaf samples in the two studies, so slight differences are to be expected. Because the environment greatly influences the capacity of a plant to retain particles, the variation in the coefficient of PM accumulation per unit area of plant leaf is generally large. This may lead to differences between the data from the present study and that of other similar research [33].

### Importance of leaf traits

**Fig 6:**
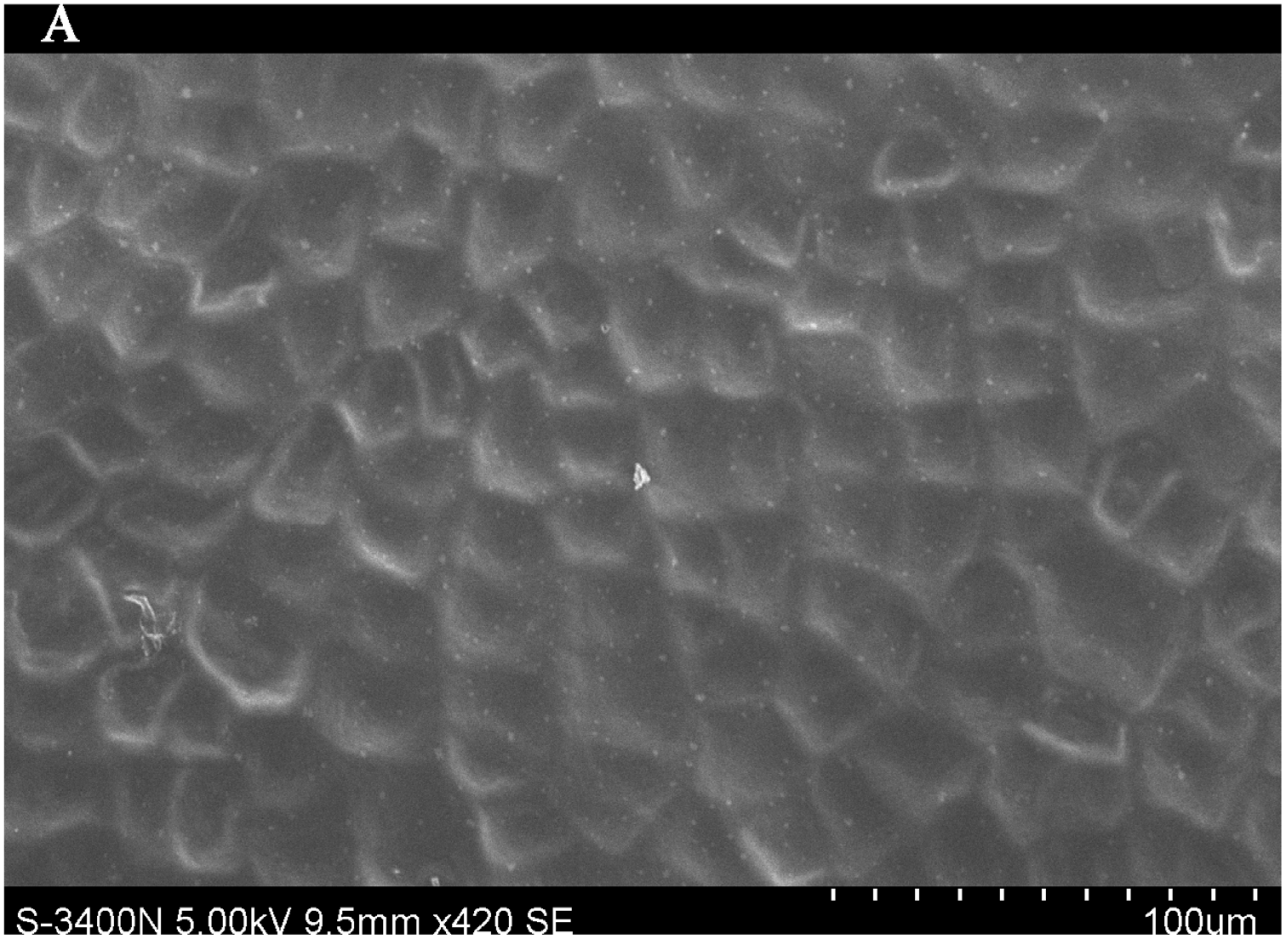
SEM photomicrographs of *Q*. *variabilis* without PM, the adaxial leave surface of *Q*. *variabilis Bl*.×420SE.

**Fig 7:**
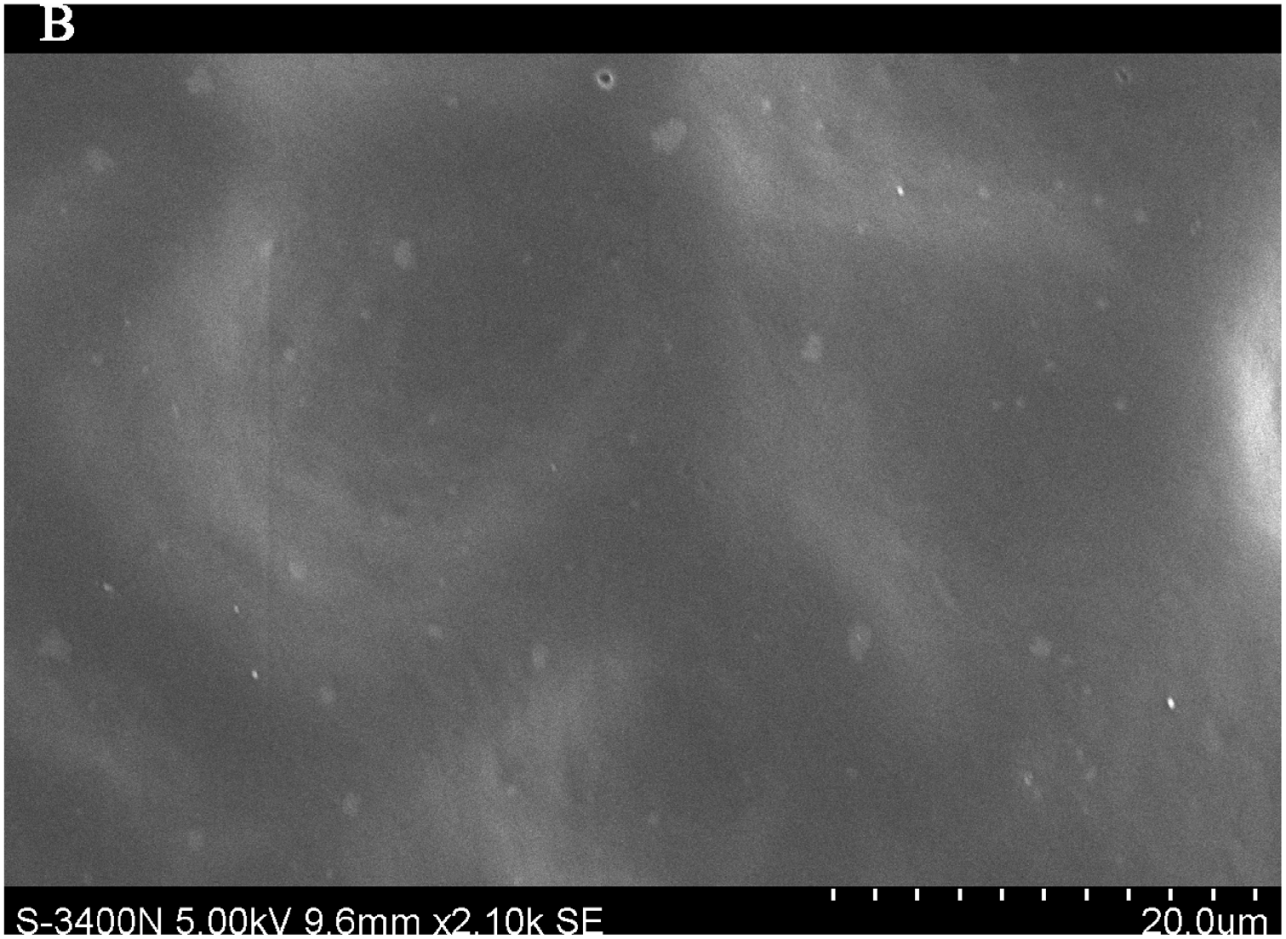
SEM photomicrographs of *Q*. *variabilis* without PM, the adaxial leave surface of *Q*. *variabilis Bl*.×2.10k SE.

**Fig 8:**
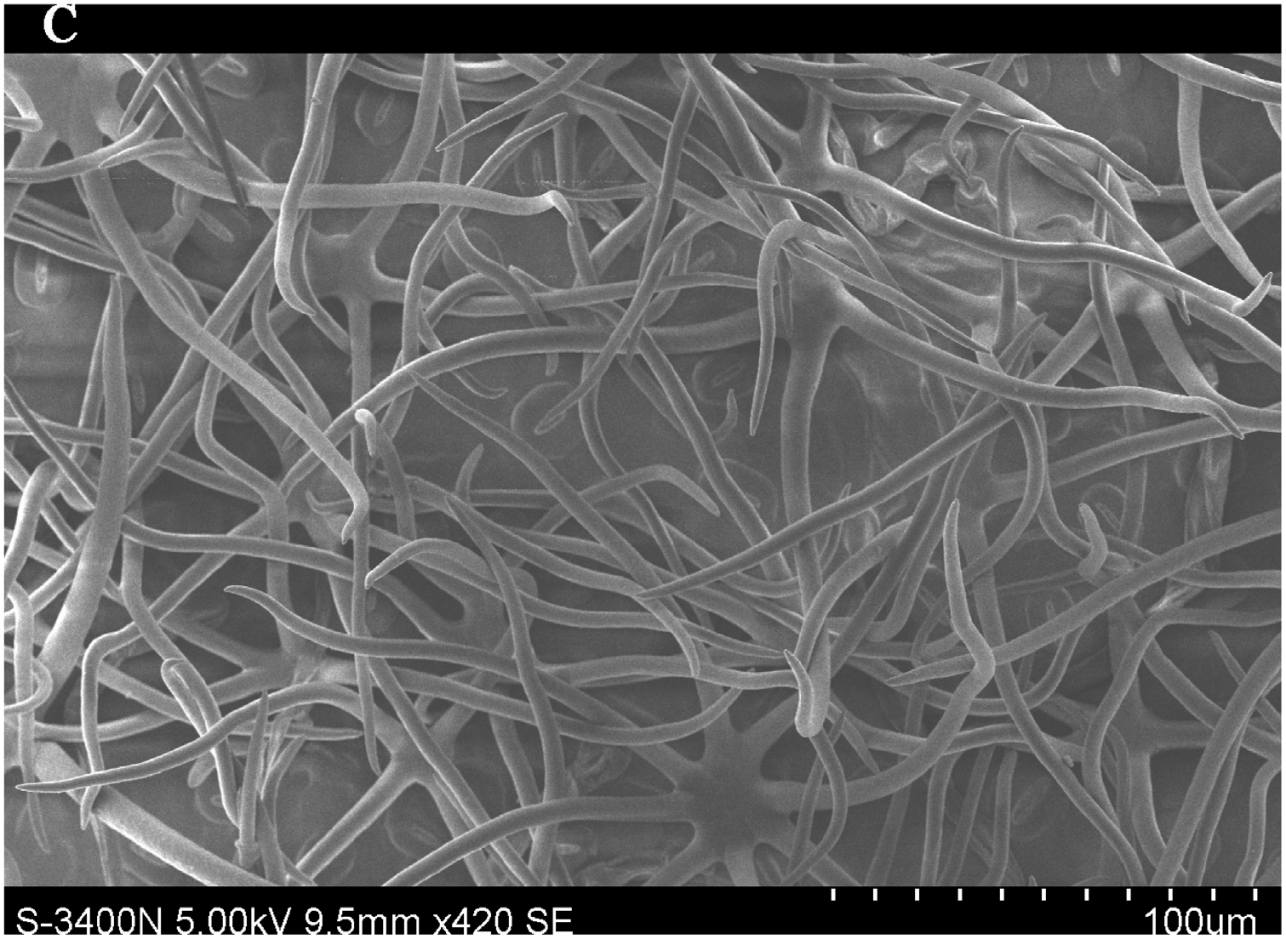
SEM photomicrographs of *Q*. *variabilis* without PM, the abaxial leaf surface of *Q*. *variabilis Bl*.×420SE.

**Fig 9:**
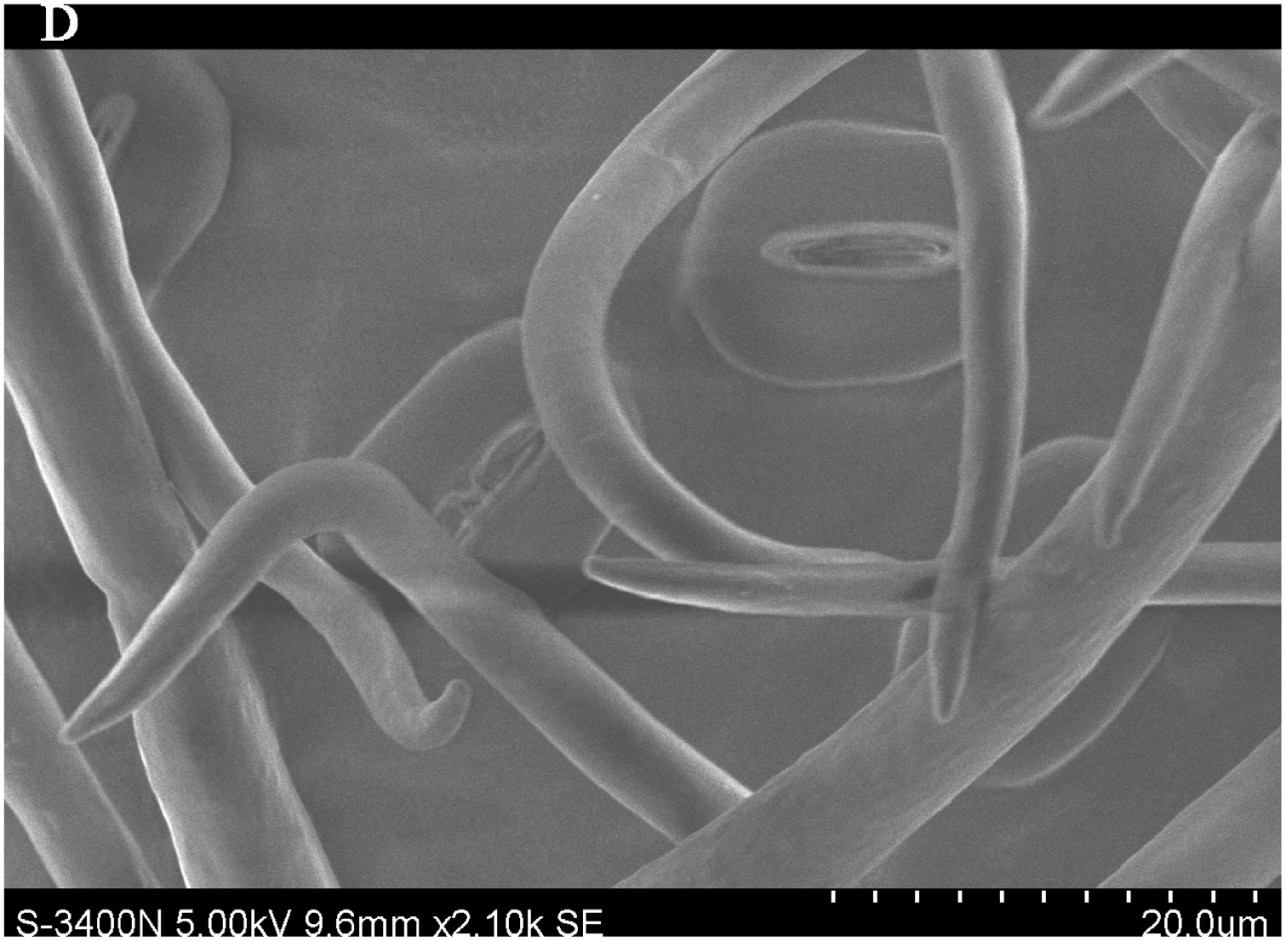
SEM photomicrographs of *Q*. *variabilis* without PM, the abaxial leaf surface of *Q*. *variabilis Bl*.×2.10k SE.

**Fig 10:**
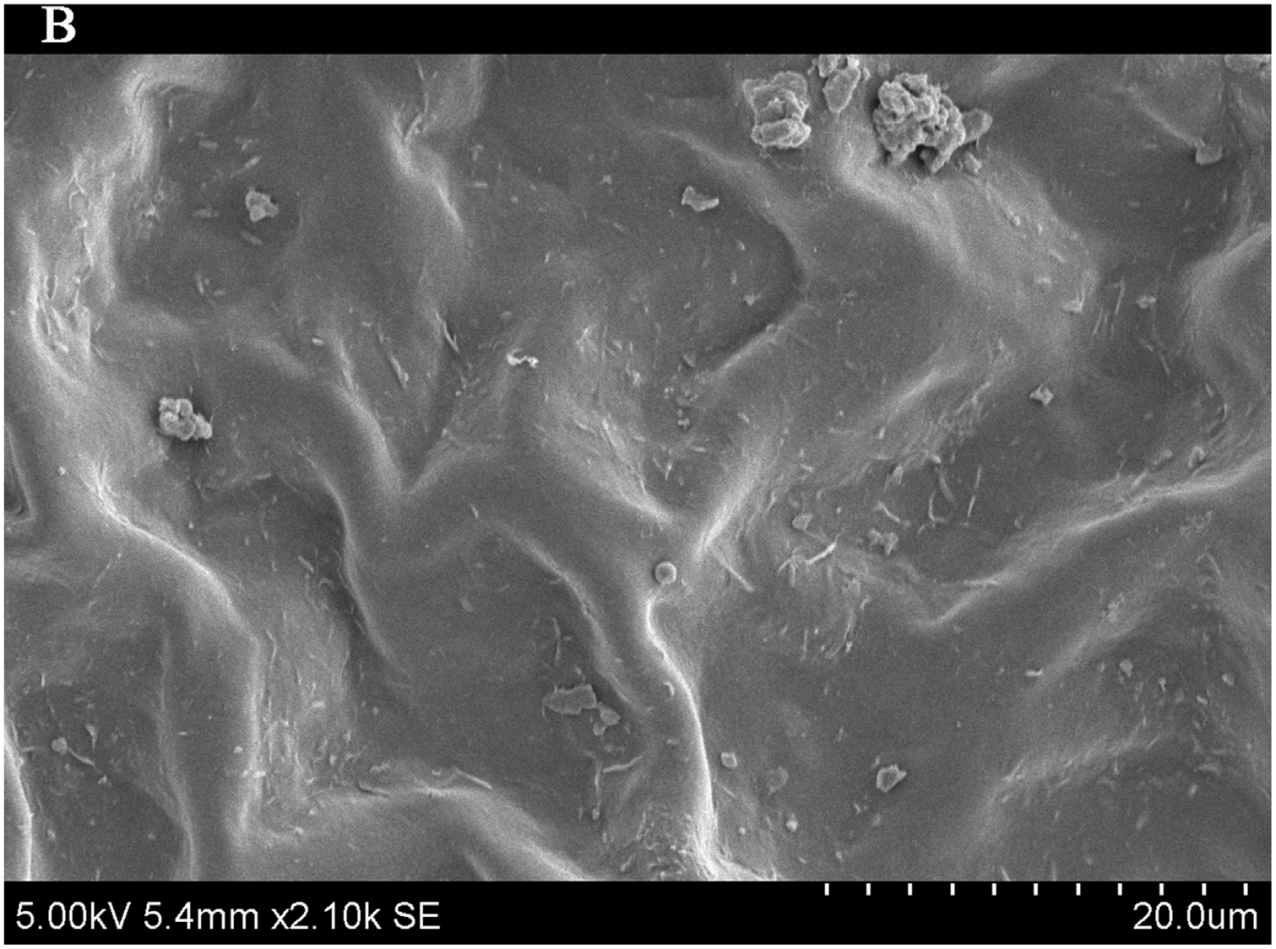
SEM photomicrographs of *Q*. *variabilis* absorbed PM, the adaxial leave surface of *Q*. *variabilis Bl*.×420SE.

**Fig 11:**
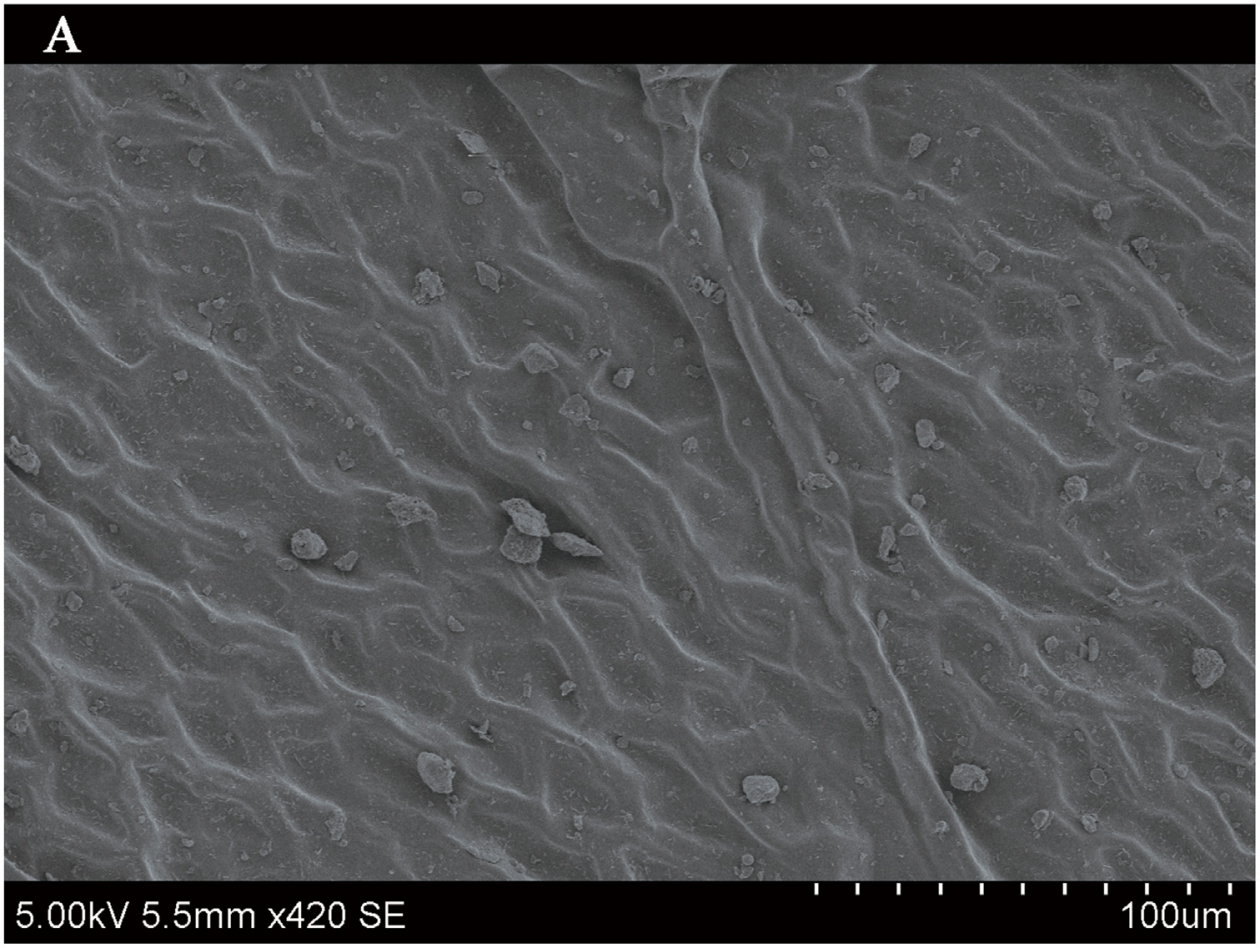
SEM photomicrographs of *Q*. *variabilis* absorbed PM, the adaxial leave surface of *Q*. *variabilis Bl*.×2.10k SE.

**Fig 12:**
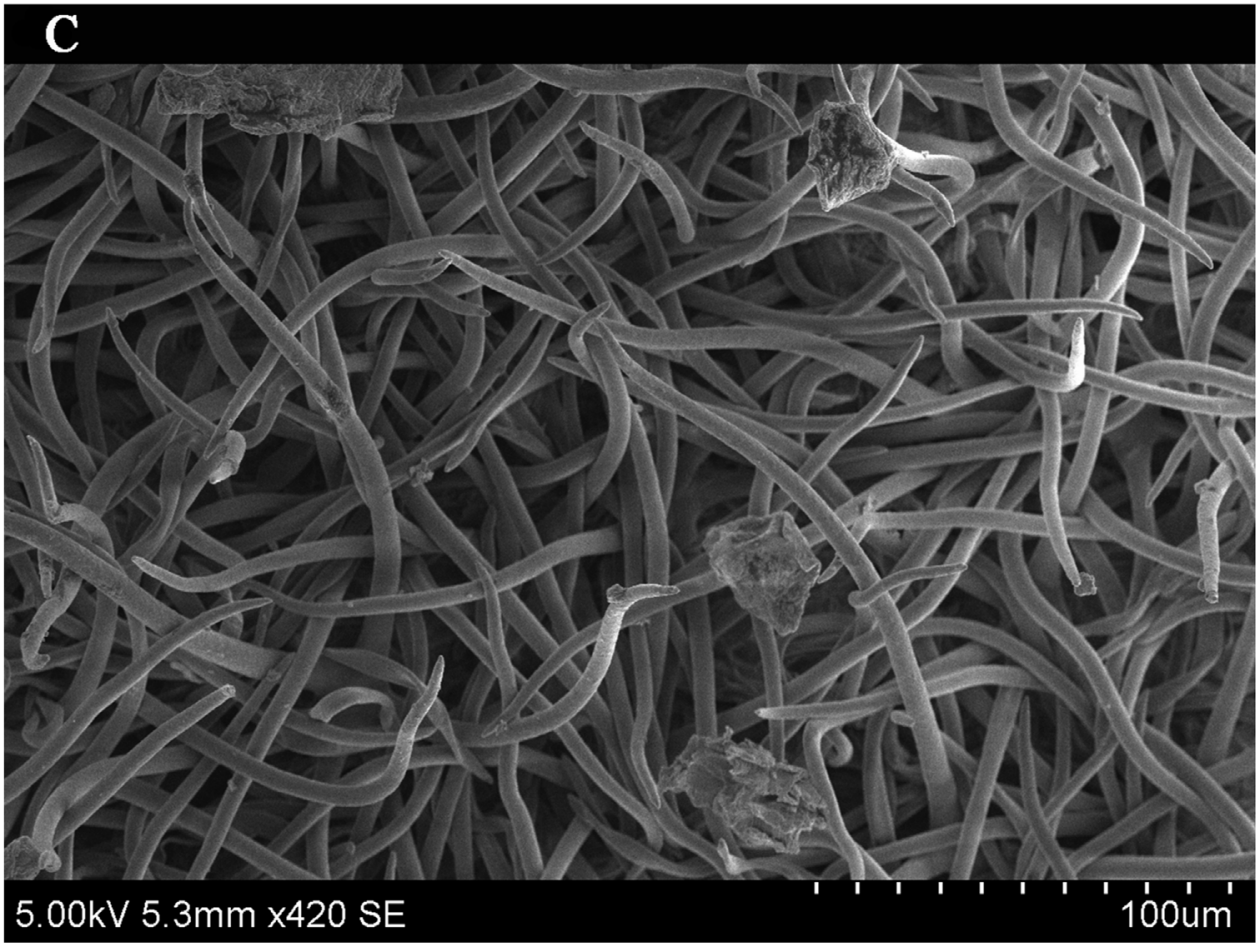
SEM photomicrographs of *Q*. *variabilis* absorbed PM, the abaxial leaf surface of *Q*. *variabilis Bl*.×420SE.

**Fig 13:**
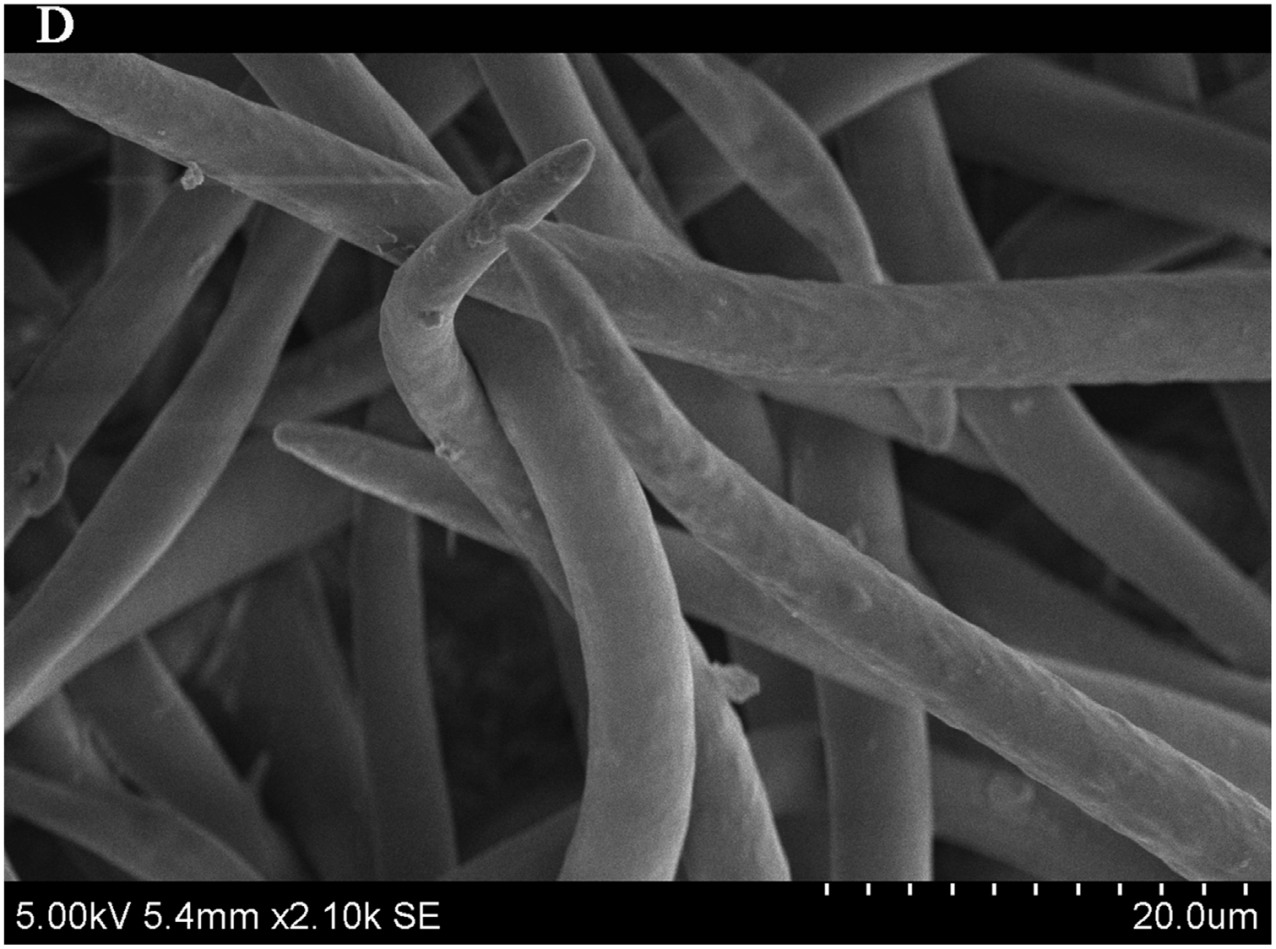
SEM photomicrographs of *Q*. *variabilis* absorbed PM, the abaxial leaf surface of *Q*. *variabilis Bl*.×2.10k SE.

The leaf blade serves as the main plant structure that captures atmospheric PM; differences in leaf surface characteristics influence the ability of different plants to retain atmospheric PM. To explore and confirm the effects of plant leaf structure on the detention of PM, we used SEM to observe typical new leaves in our plant samples including SEM photomicrographs of leaves that were not contaminated by PM (Figs 6–9) and those that were (Figs 10–13). By comparing Figs 6–9 and 10–13, the absorbed particles can be easily observed on the leaf surface. As Figs 10–13 show, many fine and coarse particles accumulated on the adaxial leave surfaces, while some large particles attached to the abaxial leaf surfaces and few particles became attached in the vicinity of stomata.

Faini (1999) found that the hydrophobicity of wax particles on leaf surface could lessen the ability of leaves to collect PM [34]. Grooves are the main parts of a blade that adsorb PM2.5. Deep grooves can intercept more particles; deeper grooves make the release of PM less likely. Shallow grooves result in only a low level of blade surface roughness and capture only a comparatively small number of particles. Broadleaf plants exhibit relatively low stomatal density; because few particles are captured near stomata, low stomatal density provides the plant with an advantage in the retention of fine particles from a human perspective. This same phenomenon was observed by SEM in this study; *Q*. *variabilis* accumulated large numbers of fine particles on its adaxial leaf surfaces [35, 36].

The accumulation of PM was different between the adaxial and abaxial leaf surfaces; this was driven by plant structure although it was not obvious using SEM. Wang et al. [28] found that only 17% of PM was deposited on the abaxial surface of the leaf. Hwang et al. [15], however, found the pubescent underside of *P*. *occidentalis* leaves were more efficient in capturing PM than the smooth adaxial side. Otteléet al. [37] proposed that wind turbulence can explain the difference between the particle counts on the adaxial and abaxial leaf surfaces. No significant difference was observed in the composition of particle sizes on both sides of a leaf for the same species, indicating that the particles were deposited on both the adaxial and abaxial leaf surfaces without separation. The ability of a blade to accumulate PM and the distribution or particle size is closely related to the leaf surface structure[38–42]. Variations in the microstructure of the adaxial and abaxial surfaces of leaves led to a difference in the quantity and quality of particles accumulated on those leaves. If a leaf has significant microstructure, such as a rough surface, pubescence and a grooved ridge protuberance, mucus oils and short petioles, it can retain large numbers of particles [43].

For plants with tomentose pubescence, a positive relationship was observed between leaf hair density and PM accumulation. Although PM10 and PM2.5 were not obviously seen on the pubescent underside of leaves, tomentose pubescence was efficient in capturing and accumulating PM. More fine particles adhered to tomentose pubescence than to other places on the leaf surface, e.g. *Kolkwitzia amabilis*, *Pyrus betulifolia* and *Q*. *variabilis*. Particles become entangled in and adhere to tomentose pubescence when contacting these pubescent surfaces. Wang et al. [25] found that leaves with low density and long needles were unfavorable for PM accumulation; Chen et al. [44] also thought the density of tomentose pubescence clearly influenced the ability of plants to accumulate PM.

Many coarse particles had become buried near the leaf folds, with many fine and ultra-fine particles attached at this location. Plant leaf folds help accumulate different size particles efficiently. Sæbø[31] and Wang et al. [45] both noted that wrinkled leaves have captured larger amounts of PM than smooth leaves. Different plants have different abilities to capture PM because of their different microscopic structure. Leaf traits and PM fraction accumulation showed a positive correlation.

## Conclusions

All plants included in the present study accumulated PM of different particle sizes (fine, 0.2–2.5 μm; coarse, 2.5–10 μm; large, 10–100 μm). PM was deposited on both the adaxial and abaxial surfaces leaves, and was trapped in leaf waxes.

Leaves with greater amounts of pubescence and rougher surfaces collected and accumulated greater amounts of PM. The adaxial leaf surfaces collected more particles than the abaxial surfaces. The most efficient shrub species for capturing PM were *C*. *sinensis* and *E*. *japonicus*; the most efficient trees were *B*. *papyrifera*, *P*. *occidentalis* and *Q*. *variabilis*. These are common species in Beijing, China. Because leaves are able to adsorb significant quantities of air pollutants, these species may be attractive options for landscaping in urban green areas. Deploying these plants more broadly as a part of urban greening efforts is feasible and environmental friendly.

## Supporting Information

S1 File Data used for analyze the average PM accumulations on leaf surface and in wax layer. It is the data of average accumulation for the PM fractions accumulated both on leaf surface and in wax layer. This data contains two parts: 1. Different sized particles accumulated on leaf surfaces 2. Different sized particles accumulated in wax layer.
(XLSX)
